# Performance Evaluation of Nano-Silica-Reinforced Mortar Containing Waste Tire Rubber and Recycled Fine Aggregate: Mechanical Properties, Frost Resistance, and Microstructure Assessment

**DOI:** 10.3390/nano15211607

**Published:** 2025-10-22

**Authors:** Yan Yan, Guofu Chen, Hang Chen, Zhukai Li

**Affiliations:** 1 School of Civil Engineering, Southwest Jiaotong University, Chengdu 610031, China; 2 School of Architectural Engineering, Guang’an Vocational and Technical College, Guangan 638000, China; 3 Collaborative Innovation Center for Building Structural Inspection and Urban Renewal, Guang’an 638000, China

**Keywords:** recycled fine aggregates, waste tire rubber, nano-silica, hydration products, compressive strength

## Abstract

In the preparation of rubber-recycled cement mortar (RRCM), recycled fine aggregates (RFA) were used to replace 95% of natural fine aggregates (NFA) by mass, with an additional 5% of NFA replaced by rubber particles (RP). Additionally, nano-silica (NS) was incorporated to replace ordinary Portland cement (OPC) by mass at a replacement of 0%, 1%, 2%, 3%, and 4%. The study aimed to investigate the effects of NS on the mechanical properties, freeze–thaw resistance, and microstructure of RRCM, using techniques such as X-ray diffraction (XRD), thermogravimetric analysis (TG-DTG), and scanning electron microscopy (SEM) to reveal the enhancement mechanisms. The results indicated that the compressive strength and flexural strength of RRCM at 28 days decreased by 10.3% and 10.1%, respectively, compared to NCM. After adding 1–3% NS, the mechanical properties of RRCM were improved, with the enhancements increasing as the NS content increased. Specifically, RRCM3 exhibited a 7.7% and 7.6% improvement in compressive and flexural strength, respectively, compared to RRCM0. After 30 freeze–thaw cycles, the strength loss rate of RCM was 27.51%, whereas the strength loss rate of RRCM3 was reduced to 20.13%, with better overall appearance integrity. Moreover, NS promoted the hydration of cement; reduced the contents of tricalcium silicate (C_3_S), and dicalcium silicate (C_2_S) and calcium hydroxide (CH); and facilitated the formation of additional hydration products that filled the interfacial transition zone (ITZ). The incorporation of 3% NS was found to provide the optimal improvement in RRCM.

## 1. Introduction

With the increasing number of urban renewal projects, construction and demolition waste (CDW) has surged dramatically, currently accounting for 30–40% of total waste generation in China [1]. The common practice of open-air stockpiling or landfilling not only occupies substantial land resources and incurs additional economic costs but also creates significant environmental pressures. Consequently, scientific and effective waste management approaches have become imperative to address this escalating challenge. The recycling of waste concrete into recycled coarse aggregate (RCA, particles ≥ 4.75 mm) for construction applications has been demonstrated as a viable solution to both resource scarcity and waste utilization problems [2]. Previous studies have confirmed that concrete incorporating RCA within certain replacement ratios can meet practical engineering requirements [3,4].

During RCA production, recycled fine aggregate (RFA, particles < 4.75 mm) is simultaneously generated. Compared to natural fine aggregate (NFA) and RCA, RFA contains higher proportions of old mortar fragments, resulting in lower strength and higher water absorption that limit its widespread application [5,6,7,8,9,10,11,12,13,14,15,16,17]. For instance, Evangelista et al. [18] found minimal negative impact on concrete strength below 30% RFA incorporation, though significant strength reduction was observed at higher replacement ratios. In addition, Munir et al. [19] indicated that when the replacement ratio of RFA reached 50%, a significant reduction in strength was observed. Additionally, some studies indicated that the use of RFA has an adverse effect on the durability of mortar or concrete. Topçu et al. [20] prepared recycled mortar by substituting quartz sand with crushed brick fine aggregate and observed that the 60-day drying shrinkage of the recycled mortar was approximately four times higher than that of natural mortar. Similarly, Ledesma et al. [21] demonstrated that the replacement rate of recycled aggregates significantly influences the permeability of mortar. With the incorporation of 40% RFA, the mortar’s resistance to penetration decreased by approximately 52%.

As the world’s largest automobile market, China produces a vast number of waste tires annually [22]. The utilization of crumb rubber particles (RP) obtained from waste tire recycling as a substitute for natural river sand in concrete preparation has been demonstrated to address several limitations of recycled mortar, including excessive self-weight and poor ductility, while simultaneously expanding the application fields of waste tires [23,24]. Previous research revealed that incorporating RP into recycled aggregate concrete improved its workability, with concrete fluidity progressively enhancing as the RP content increased [25,26]. Segre et al. [27] conducted experimental studies on the frost resistance of RP mortar, finding that mortar containing 10% RP exhibited only a 20% reduction in flexural strength after 60 freeze–thaw cycles, compared to a 75% reduction observed in conventional mortar. However, the organic nature of RP resulted in poor bonding with cementitious materials and lower strength compared to RFA, leading to inferior mechanical properties of rubber-recycled cement mortar (RRCM) [28,29]. These limitations have constrained the utilization of both RP and RFA in civil engineering applications.

To address these deficiencies, supplementary cementitious materials have been investigated for RRCM performance enhancement. Recent advancements in nanotechnology have enabled the production of highly reactive nanoparticles with superior properties, facilitating their broader application in civil engineering [30,31]. Among various nanomaterials, nano-silica (NS) demonstrates exceptional pozzolanic activity, nucleation effects, and filling capability [32]. When incorporated into cementitious materials, NS promotes cement hydration, fills internal pores, and reacts with calcium hydroxide to form calcium silicate hydrate gel, thereby improving the hardened material’s strength [33,34,35,36,37,38,39,40]. Mei et al. [41] found that the ultrafine particle size of NS enabled it to fill nanoscale pores in cement mortar, increasing the material density. Additional studies confirmed that NS accelerates cement hydration and improves both the compressive and flexural strength of concrete [42]. However, some studies have indicated that nano-silica (NS) can adversely affect the workability of cement-based materials [43,44,45]. These findings underscore the engineering significance of investigating NS’s effects on RRCM performance.

This study examines RRCM prepared with 95% RFA and 5% RP replacements of natural river sand, incorporating NS at varying dosages (0%, 1%, 2%, and 3%). Comprehensive evaluations of workability, mechanical properties, and frost resistance were conducted. Advanced characterization techniques including scanning electron microscopy (SEM), X-ray diffraction (XRD), and thermogravimetric analysis (TG-DTG) were employed to analyze microstructural evolution and hydration product formation in RRCM.

## 2. Materials and Methods

### 2.1. Raw Materials

**Figure 1:**
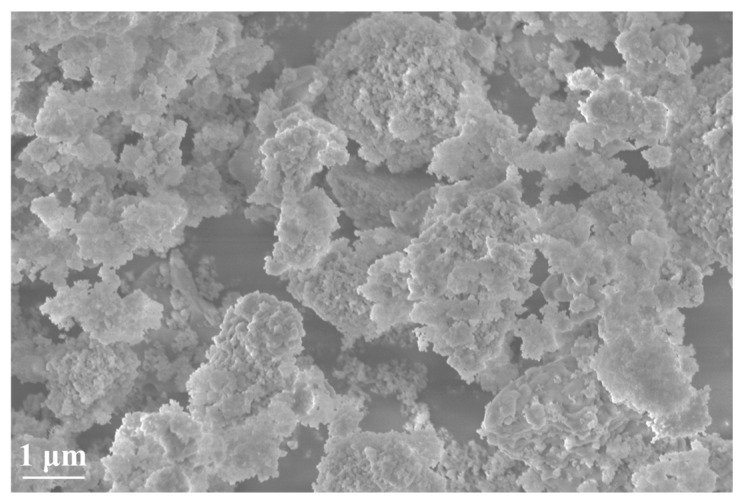
SEM images of NS.

The materials required for this experiment include P.O 42.5 ordinary Portland cement, natural fine aggregates (NFA), recycled fine aggregates (RFA), rubber particles (RP), and nano-silica (NS). The performance specifications of the cement are shown in Table 1. Quartz sand was used as the NFA, while RFA was sourced from local house demolitions in Guang’an City, obtained through the processes of the crushing, screening, and drying of waste concrete. The physical properties NFA and RFA are presented in Table 2. The RP were provided by a local discarded tire processing plant, with a particle size ranging from 1 to 2 mm, and they had not undergone any other treatment. The NS exhibited a particle size ranging from 30 to 55 nm, with a specific surface area of 540 m^2^/g. Its electron microscope is shown in Figure 1: the NS was flocculent and had many micropores.

### 2.2. Mix Design and Preparation

Seven groups of cement mortar specimens were prepared. NS replaced cement at mass replacement ratios of 1%, 2%, 3%, and 4%, while RFA replaced 95% of NFA and RP replaced 5% of NFA by mass. These mixtures resulted in three categories: natural cement mortar (NCM), recycled cement mortar (RCM), and rubber-recycled cement mortar (RRCM). The mix proportions are shown in Table 3, and RRCMx indicates RRCM with x% NS (x = 1–4).

**Figure 2:**
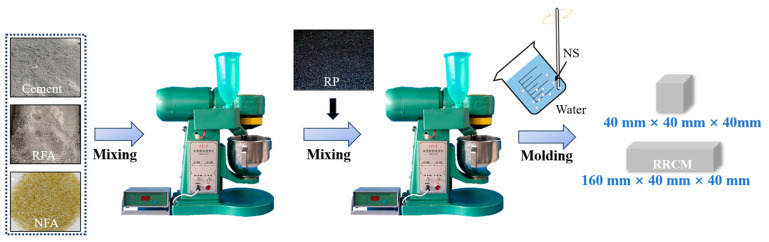
Mixing method of RRCM.

The production process of RRCM is shown in Figure 2. The preparation procedure consisted of the following steps: (1) Mixing cement, RFA, and NFA in a mixer for 1 min; (2) adding RP and mixing for 30 s; (3) NS was mixed with water and stirred using an ultrasonic disperser (LD-CP150, Leander, China) at a frequency of 20 Hz for 5 min to form a suspension, and then this suspension was incorporated into the mixer and mixed for 3 min to obtain a homogeneous mortar mixture; (4) the mixture was cast into molds, demolded after 1 day, and cured in a standard curing room. Two specimen sizes were prepared: 40 mm × 40 mm × 160 mm for flexural strength testing and 40 mm × 40 mm × 40 mm for compressive strength, water absorption, and frost resistance measurements.

### 2.3. Test Methods

#### 2.3.1. Fluidity

According to the Chinese standard GB/T 2419–2005 [46], the mortar mixture was divided into two layers and loaded into a test mold. It was vibrated twice on a jumping table, and the longitudinal and transverse diameters of the mixture were measured. The average value was taken as the fluidity of the sample.

#### 2.3.2. Mechanical Properties

In accordance with GB/T 17671–2023 [47], the flexural and compressive strengths of all specimens were measured using a universal testing machine (WDW-300, Taylor, China) after 7 and 28 days of curing. The loading rates were maintained at 50 ± 10 N/s for flexural tests and 2.40 kN/s for compressive tests. For each group, three replicates were tested, and the average value was adopted as the final result.

#### 2.3.3. Water Absorption Test

After drying the specimens to a constant weight in a 40 °C chamber (XD-1AII, Xundi, China), the water absorption test was conducted. The specimens were soaked in water for 24 h to test their water absorption. Three specimens were tested in each group, and the average value was taken as the test result for that group.

#### 2.3.4. Frost Resistance Test

The freeze–thaw resistance of specimens cured for 28 days was evaluated in accordance with JGJ/T 70–2009 [48]. The specimens were fully submerged in water using a freeze–thaw testing chamber (DX-170-40, Huasheng, China) and subjected to 30 cycles, each comprising a freezing phase at −18 ± 2 °C for 16 h followed by thawing in a 25 ± 2 °C water bath for 6 h. Mass and compressive strength measurements were conducted after the 10th, 20th, 25th, and 30th cycles to determine the mass loss rate and strength loss rate.

#### 2.3.5. XRD

The hydration products of RRCM were analyzed by X-ray diffractometer (D8 ADVANCE, Bruker AXS, Germany), using a Cu target and testing conditions of 40 kV, 30 mA, scanning angle of 5~60°, and scanning speed of 5 °C/min.

#### 2.3.6. TG-DTG

In order to calculate the content of the hydration products of the RRCM, the STA499C thermogravimetric analyzer (STA499C, NETZSCH, Germany) was used to test the mass loss of the sample in various temperature ranges. During the testing process, nitrogen gas was used as a protective gas, and the heating rate was 10 °C/min. The test temperature was between 30 °C and 800 °C.

#### 2.3.7. SEM

The microstructure of the specimen after curing for 28 days was observed under vacuum using a scanning electron microscope (Gemini SEM 300, ZEISS, Germany).

## 3. Results

### 3.1. Fluidity

**Figure 3:**
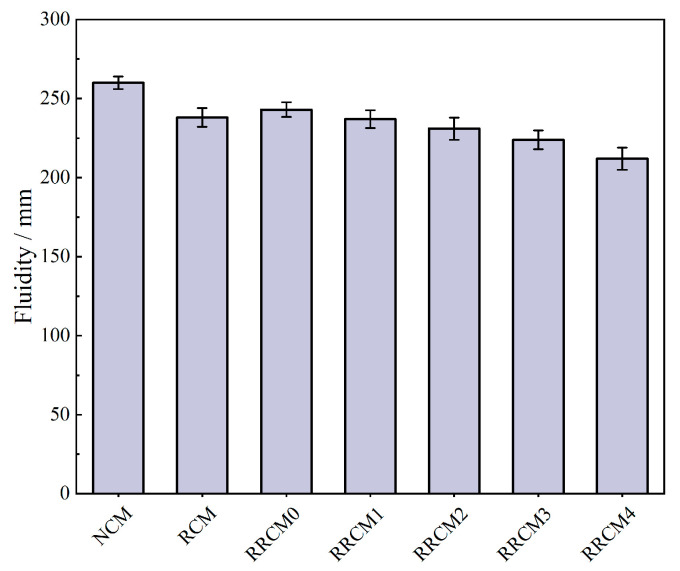
Fluidity of different types of mortars.

Figure 3 reveals the fluidity of the mortar. The fluidity of NCM is 260 mm, while the fluidity of RCM decreases to 238 mm, representing an 8.5% reduction. This reduction is attributed to the high water absorption and rough surface texture of RFA, which lower the effective water-to-cement ratio within the mortar, increasing the cohesiveness of the RCM [6]. The incorporation of RP increases the fluidity of RRCM0 to 243 mm, which is attributed to the hydrophobic of RP, leading to an increase in free water in the RCM mixture. Additionally, the action of RP helps form a rolling effect inside the concrete mixture, which reduces the friction between the components [25]. After adding NS, the fluidity of RRCM shows a downward trend, with RRCM4 being lower than RRCM0. This can be attributed to the smaller particle size of NS, which has an extremely high specific surface energy [45]. It has a strong adsorption capacity for free water in the mixture, resulting in an increase in viscosity and a decrease in fluidity.

### 3.2. Compressive Strength and Flexural Strength

**Figure 4:**
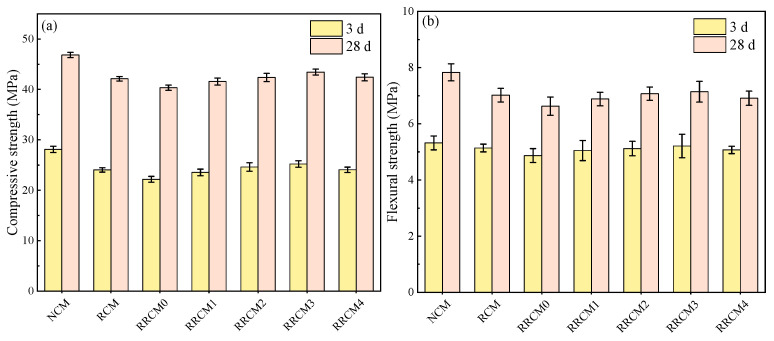
(**a**) Compressive strength and (**b**) flexural strength of different types of mortars.

Figure 4 presents the compressive strength and flexural strength of specimens. After the incorporation of RFA and RP, the compressive strength of the mortar at all ages decreases by 13.9% to 21.1%, while the flexural strength decreases by 8.5% to 15.3%. However, after adding NS, both the compressive and flexural strengths of RRCM show varying degrees of improvement, particularly in the early strength. Following the incorporation of 3% NS, the 3-day compressive strength of RRCM3 increases by 13.7%, and the flexural strength increases by 7.0% compared to RRCM0. After a curing period of 28 days, the compressive strength and flexural strength of RRCM3 improve by 7.7% and 7.6%, respectively, compared to RRCM0. The incorporation of RFA and RP result in a decrease in the compressive and flexural strengths of RRCM. This is due to the presence of a large amount of old mortar fragments in RFA, which leads to lower inherent strength compared to natural fine aggregates (NFA) [6]. RP, being an organic material, has poor adhesion to the cement mortar, and its elastic modulus is significantly lower than that of the cement paste [27]. As a result, when RRCM is subjected to compressive loading, cracks tend to develop at the interface between RP and the mortar, leading to rapid crack propagation and specimen failure. The addition of NS accelerates the hydration rate of cement and reacts with calcium hydroxide (CH) crystals, producing additional hydrated calcium silicate (C-S-H) gel, which enhances the density of the sample structure [39]. Furthermore, the incorporation of NS significantly increases the viscosity of the cement paste, enabling the low-density RP particles to disperse more evenly throughout the cement matrix, thereby improving the hardened strength of the RRCM.

### 3.3. Flexural–Compressive Ratio

**Figure 5:**
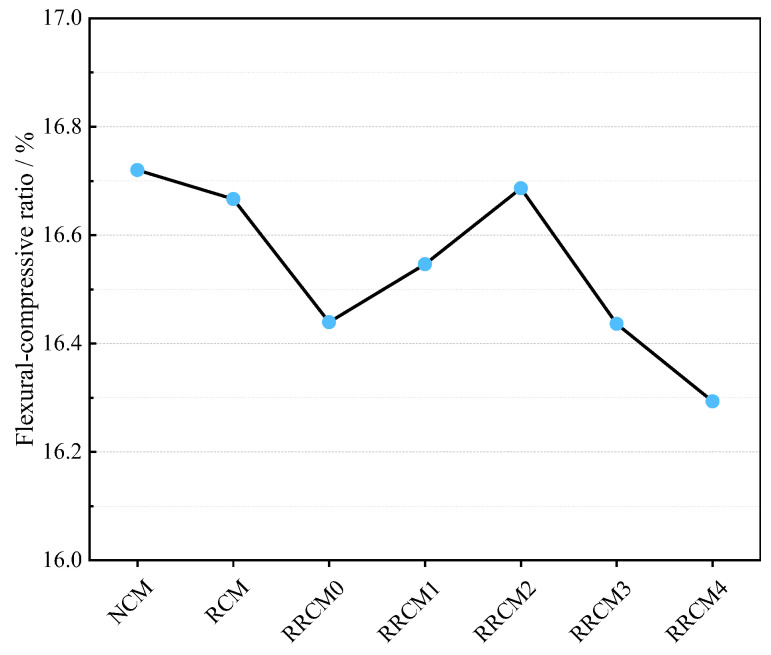
The flexural–compressive strength ratio of different types of mortars.

The flexural–compressive strength ratio is the ratio of flexural strength to compressive strength, and a higher ratio signifies greater toughness of the concrete. As shown in Figure 5, the flexural–compressive ratio of RRCM decreases after the incorporation of RFA and RP. This reduction is attributed to the presence of old mortar in RFA, which creates multiple weak interfacial transition zones (ITZ) within the RCM [7]. Additionally, the interface between RP and cement mortar forms another weak zone in RRCM, further reducing its ductility. With the addition of NS, the flexural-to-compressive strength ratio increases, and at a 2% incorporation level, the ductility and crack resistance of RRCM approach those of NCM. This improvement is due to the ability of NS to strengthen the ITZ within RRCM, enhancing the bonding between RFA, RP, and the cement matrix. However, it is noteworthy that when the NS content exceeds 2%, the flexural-to-compressive strength ratio of RRCM rapidly decreases. This may be because NS has a high specific surface energy, and when the content surpasses a certain threshold, an “agglomeration effect” occurs, preventing its even dispersion within the cement mortar and consequently reducing the ductility of RRCM [45].

### 3.4. Water Absorption

**Figure 6:**
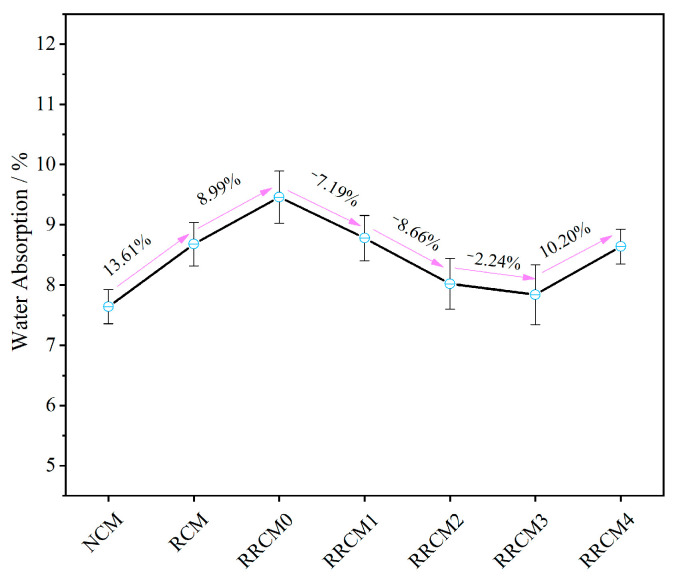
The water absorption rates of different types of mortars.

Figure 6 reflects the 24 h water absorption rates of each specimen. The 24 h water absorption rate of NCM is 7.64%. After the incorporation of RFA, the water absorption rate increases by 13.61%. Upon further incorporation of RP, the water absorption rate of RRCM0 increases by 8.99% compared to RCM. With the addition of NS, the water absorption rate initially decreases and then increases, reaching the lowest point at 7.84% when the dosage is 3%. It is evident that the high water absorption rate of RFA adversely affects the impermeability of RCM [10]. Additionally, RP acts as an air-entraining agent, introducing air bubbles during mixing and generating a significant number of pores within the cement mortar, thereby further diminishing the impermeability of RCM [45]. The small particle size of NS allows it to fill the pores between RFA and the cement matrix. Its “nucleating effect” can adsorb hydration products to repair these pores, promoting a reduction in porosity and an increase in density within the cement matrix, thus enhancing the impermeability of RRCM [34].

### 3.5. Frost Resistance

**Figure 7:**
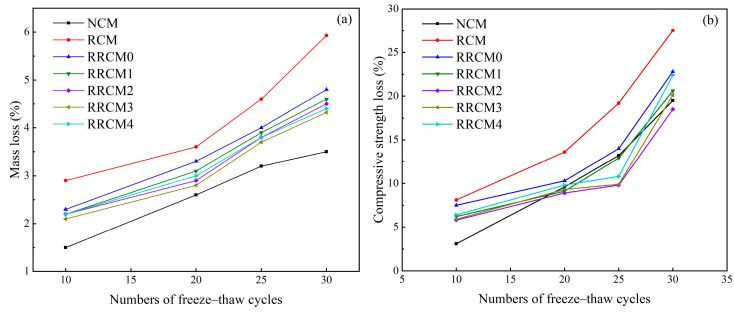
(**a**) The mass loss and (**b**) strength loss rates of different types of mortars.

Figure 7 presents the mass loss and strength loss rates of the mortar after different freeze–thaw cycles. With the increase in freeze–thaw cycles, both the mass loss and strength loss of the mortar continually increase, with RCM exhibiting the largest loss. The incorporation of RP and NS enhances the freeze–thaw resistance of RCM: after 30 freeze–thaw cycles, the mass loss and strength loss rates of RCM are 5.93% and 27.51%, respectively. When RP and 3% NS are added, the mass loss and strength loss rates decrease to 4.32% and 20.13%, respectively. Increasing the NS content beyond this level does not provide further improvement in the mortar’s freeze–thaw resistance. The pores in RFA are more numerous than those in NFA, and when water enters these pores and freezes, the resulting expansion increases the number of cracks within the matrix [10]. RP, with its good flexibility and elasticity, can alleviate the volume changes caused by freezing and thawing, thus reducing mortar damage [49,50]. The addition of NS reduces the number of harmful pores within the mortar and disrupts the interconnected pore network, significantly improving the density of the mortar and further enhancing the freeze–thaw resistance of RRCM.

**Figure 8:**
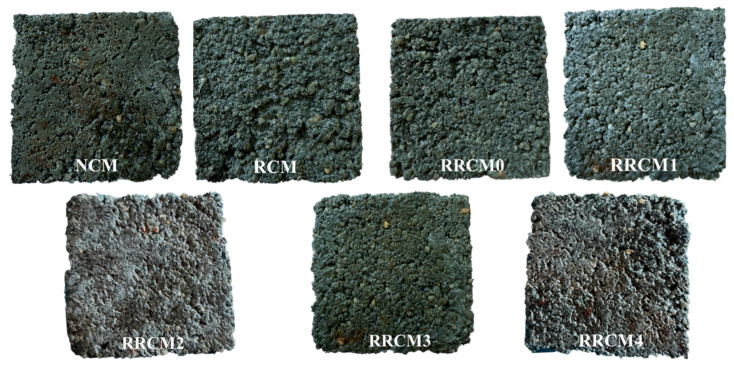
The appearance of different types of mortars after 30 freeze–thaw cycles.

Figure 8 shows the appearance of different types of mortars after 30 freeze–thaw cycles, after 30 freeze-thaw cycles, the surface of each mortar sample exhibited varying degrees of damage. The surface of NCM remained relatively smooth, with only slight aggregate exposure and detachment, and the edges of the specimen were clearly defined. In contrast, the surface of RCM showed defects, with extensive aggregate detachment. After incorporating RP, the aggregate detachment in RRCM0 was significantly alleviated. With the addition of NS, the appearance of RRCM was more intact, with fewer pits, and the effect was most pronounced when the NS content was 3%.

### 3.6. XRD and TG-DTG

**Figure 9:**
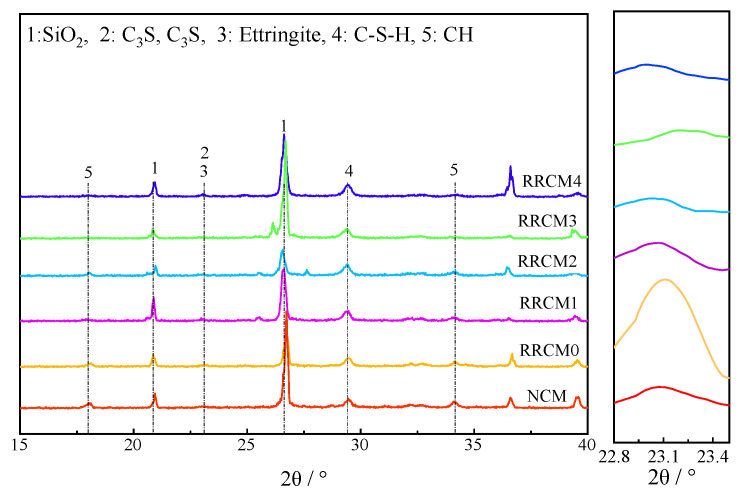
The XRD patterns of different types of mortars at 28 days.

Figure 9 displays the XRD diffraction patterns of the mortar at 28 days. Characteristic diffraction peaks of ettringite (AFt), CH, tricalcium silicate (C_3_S), and dicalcium silicate (C_2_S) are present in the mortar. Following the incorporation of RFA and RP, the peak intensities of C_3_S and C_2_S exhibit a noticeable increase. In contrast, the addition of NS leads to a reduction in the peak intensities of both phases. This is mainly due to the high pozzolanic activity of NS, which promotes the hydration of C_3_S and C_2_S [34]. The peak value of CH exhibits a slight reduction, which can be attributed to the reaction between NS and CH, leading to the formation of additional C-S-H gel.

**Figure 10:**
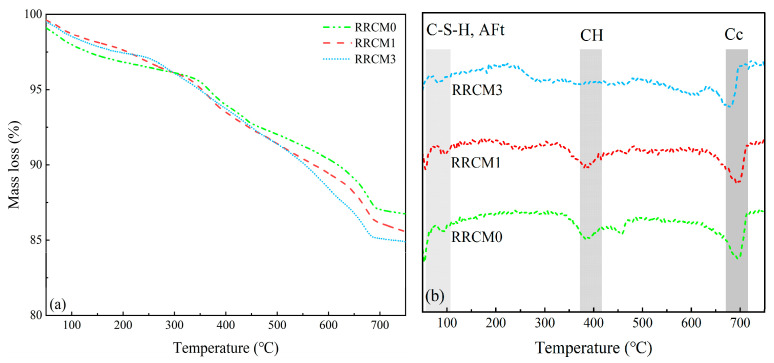
(**a**) TG and (**b**) DTG curves of different types of mortars at 28 days.

Figure 10 shows the TG-DTG curves for RRCM0, RRCM1, and RRCM3. As illustrated in Figure 10a, with an increasing dosage of NS, the total mass loss gradually increases. The endothermic peak near 400 °C observed in RRCM diminishes with the addition of NS, indicating a reduction in the quantity of CH crystals. In Figure 10b, the three peaks of the DTG curve correspond to the dehydration or decomposition of different hydration products, with detailed values provided in Table 4. It can be observed that RRCM3 exhibits a higher degree of hydration. As the NS substitution rate increases, the content of CH in RRCM gradually decreases, which is consistent with the trends observed in the XRD phase analysis. This indicates that NS can react with CH crystals to reduce their content. Generally, a lower CH crystal content in cement paste corresponds to a higher C-S-H gel content, which is beneficial for the strength development of the mortar.

### 3.7. SEM

**Figure 11:**
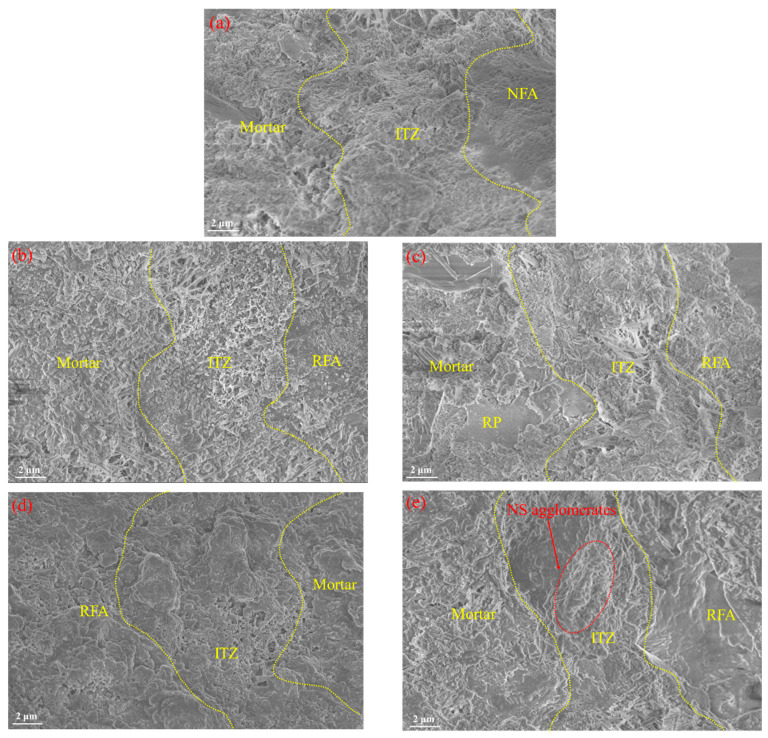
The SEM images of different types of mortars at 28 days. (**a**) NCM; (**b**) RRCM0; (**c**) RRCM1; (**d**) RRCM3; (**e**) RRCM4.

Figure 11 shows the SEM images of the mortar. As seen in Figure 11a and 11b, after incorporating RFA and RP, RRCM0 exhibits noticeable pores in the ITZ, with loosely packed hydration products. A comparison with Figure 11c reveals that the incorporation of NS promotes more hydration products to fill the ITZ. As the NS content increases, the improvement in the ITZ initially increases and then decreases. This is because NS, with its small particle size, has a “filling effect”, allowing it to fill pores and cracks. Through the “nucleation effect”, it adsorbs hydration products to repair these pores and cracks, significantly enhancing the densification of the ITZ [34]. Additionally, by promoting the hydration reaction of the new mortar, NS strengthens the bond of the new mortar. Furthermore, as seen in Figure 11d and 11e, NS plays a “bridging” role between RFA and the mortar, guiding the formation of C-S-H gel and enhancing the bond between the two. However, when the NS content reaches 4%, it agglomerates form, reducing the performance of RRCM.

## 4. Conclusions

In this work, rubber-recycled cement mortar (RRCM) was prepared by replacing natural river sand with recycled fine aggregate (RFA) and recycled powder (RP). The effects of varying amounts of nano-silica (NS) on the fluidity, mechanical properties, and frost resistance of RRCM were investigated. Additionally, the underlying mechanisms were explored using X-ray diffraction (XRD), thermogravimetric analysis (TG-DTG), and scanning electron microscopy (SEM). The main conclusions are as follows:

(1) The incorporation of RFA reduces the flowability of natural cement mortar (NCM), while the addition of RP improves the flowability of recycled cement mortar (RCM) from 238 mm to 243 mm. After incorporating RFA and RP, the compressive strength of RRCM at different curing ages decreases by 13.9–21.1%, and the flexural strength decreases by 8.5–15.3%.

(2) The addition of NS significantly improves the mechanical properties and ductility of RRCM. As the NS content increases, the improvement in strength becomes more pronounced. Compared with RRCM0, the compressive strength and flexural strength of RRCM3 have increased by 13.7% and 7.0%, respectively. When the NS content exceeds 3%, the improvement effect is no longer significant.

(3) After 30 freeze–thaw cycles, RCM experiences significant aggregate exposure and detachment, with mass loss and strength loss rates of 5.93% and 27.51%, respectively. The incorporation of RP and NS enhances the freeze–thaw resistance of RCM. The mass loss rate and strength loss rate of RRCM3 are 4.32% and 20.13%, respectively, and its appearance is relatively more complete compared to RCM.

(4) NS promotes the hydration of tricalcium silicate (C_3_S) and dicalcium silicate (C_2_S), and reacts with CH crystals to form additional hydrated calcium silicate (C-S-H) gel, which fills the pores in RRCM. Furthermore, NS adsorbs hydration products and repairs cracks in the weak interfacial transition zone (ITZ), improving the performance of the weak ITZ within RRCM and increasing the densification of the cement matrix.

NS can promote the hydration of C_3_S and C_2_S and undergo a secondary hydration reaction with CH crystals to generate additional C-S-H gels, filling the pores inside RRCM. Moreover, NS can adsorb hydration products to repair cracks inside weak ITZ, improve the performance of weak ITZ inside RRCM, and thereby enhance the compactness of the cement matrix.

This study improved the engineering performance of RRCM by incorporating NS as an auxiliary cementitious material, which increased the amount of waste concrete and waste tires in cement-based materials, and saved a significant amount of NFA. In China, industrial-grade NS costs approximately USD 7/ton, while natural sand is priced at about USD 30/ton. Since RFA can be obtained at virtually no cost, and considering the very small dosage of NS, this research demonstrates a strategy for achieving significant economic and environmental benefits. Nevertheless, the current investigation primarily addresses variations in mechanical properties, and further research is required to evaluate the durability performance of such materials.

## Figures and Tables

**Table 1:** Basic physical and mechanical properties of cement.

Stability	Setting Time/min		Compressive Strength/MPa		Flexural Strength/MPa	
	Initial	Final	3 d	28 d	3 d	28 d
Qualified	203	298	22.1	47.8	5.4	7.5

**Table 2:** Physical properties of the NFA and RFA.

Type	Apparent Density/kg·m^3^	Water Absorption/%	Fineness Modulus
NFA	2680	1.80	2.21
RFA	2468	6.50	2.34

**Table 3:** Mixed proportions.

Type	Raw Material Consumption (kg/m^3^)					
	Cement	NS	NFA	RFA	Water	RP
NCM	450	0	1350	0	225	0
RCM	450	0	0	1350	225	0
RRCM0	450	0	0	1269	225	81
RRCM1	445.5	4.5	0	1269	225	81
RRCM2	441	9	0	1269	225	81
RRCM3	436.5	13.5	0	1269	225	81
RRCM4	432	18	0	1269	225	81

**Table 4:** The content of different hydration products of different types of mortars.

Specimen	CH (%)	Bounding Water (%)
RRCM0	10.89	9.94
RRCM1	10.42	10.56
RRCM3	8.54	10.65

## Data Availability

The data presented in this study are available on request from the corresponding author.
